# Understanding Suicide in Our Community through the Lens of the Pediatric ICU: An Epidemiological Review (2011–2017) of One Midwestern City in the US

**DOI:** 10.3390/children8020059

**Published:** 2021-01-20

**Authors:** Andrew Kampfschulte, Matthew Oram, Alejandra M. Escobar Vasco, Brittany Essenmacher, Amy Herbig, Aniruddh Behere, Mara L. Leimanis-Laurens, Surender Rajasekaran

**Affiliations:** 1 Office of Research and Education, Spectrum Health, 15 Michigan Street NE, Grand Rapids, MI 49503, USA; andrew.kampfschulte@spectrumhealth.org (A.K.); surender.rajasekaran@spectrumhealth.org (S.R.); 2 Department of Pediatrics and Human Development, College of Human Medicine, Michigan State University, Life Sciences Building, 1355 Bogue Street, East Lansing, MI 48824, USA; orammatthew1@gmail.com (M.O.); alejandra.escobarvasco@spectrumhealth.org (A.M.E.V.); amy.herbig@spectrumhealth.org (A.H.); aniruddh.behere@helendevoschildrens.org (A.B.); 3 Pediatric Intensive Care Unit, Helen DeVos Children’s Hospital, 100 Michigan Street NE, Grand 16 Rapids, MI 49503, USA; brittany.essenmacher@spectrumhealth.org; 4 Pediatric Behavior Health, Helen DeVos Children’s Hospital, 100 Michigan Street NE, Grand 14 Rapids, MI 49503, USA

**Keywords:** suicide, pediatrics, critical care, geospatial, temporal, self-directed violence

## Abstract

Suicide frequency has tripled for some pediatric age groups over the last decade, of which, serious attempts result in pediatric intensive care unit (PICU) admissions. We paired clinical, aggregate geospatial, and temporal demographics to understand local community variables to determine if epidemiological patterns emerge that associate with risk for PICU admission. Data were extracted at an urban, high-volume, quaternary care facility from January 2011 to December 2017 via ICD 10 codes associated with suicide. Clinical, socioeconomic, geographical, and temporal variables were reviewed. In total, 1036 patients over the age of 9 were included, of which *n* = 161 were PICU admissions. Females represented higher proportions of all suicide-related hospital admissions (67.9%). Looking at race/ethnicity, PICU admissions were largely Caucasian (83.2%); Blacks and Hispanics had lower odds of PICU admissions (OR: 0.49; 0.17, respectively). PICU-admitted patients were older (16.0 vs. 15.5; *p* = 0.0001), with lower basal metabolic index (23.0 vs. 22.0; *p* = 0.0013), and presented in summer months (OR: 1.51, *p* = 0.044). Time-series decomposition showed seasonal peaks in June and August. Local regions outside the city limits identified higher numbers of PICU admissions. PICUs serve discrete geographical regions and are a source of information, when paired with clinical geospatial/seasonal analyses, highlighting clinical and societal risk factors associated with PICU admissions.

## 1. Introduction

Suicide is defined by the Centers for Disease Control as death caused by injuring oneself with the intent to die and is part of a broader class of behaviors called self-directed violence, that is a behavior that could result in immediate injury and has potential for lasting injury [1]. A serious suicide attempt (SSA) is one that would result in death without specialized intervention (surgery, antidotes, intensive care unit (ICU) admission, prolonged hospitalization), and can be considered a proxy for completed suicide in surviving individuals [2]. This further breakdown of patients into two groups may be summarized as: those requiring hospitalization for observation versus those facing potential injury or risk of mortality, thus requiring an ICU admission.

Suicide is a major and ongoing public health concern in the United States (US). Current data demonstrate that death by suicide is the second leading cause of mortality in people aged 15–24 years [3,4]. Suicide affects young people from all races and socioeconomic groups. Rates of suicide have increased since the turn of the century in both adults and children [3]. From 1999–2014, the rate of suicide for children age 10–14 tripled, helping to drive the overall increase in rate of suicide across all ages during the same time period [5].

Children’s hospitals serve distinct geographical areas, where patients needing highly advanced care are sent to their regional ICU. We are the only pediatric ICU (PICU) in a city of above 200,000 residents with a catchment area that includes one million residents. Patients that originate from these regions are being exposed to a unique set of geographical and temporal factors that need better understanding and characterization.

In this retrospective chart review, we extracted clinical and community variables available in the electronic medical record (EMR) to develop a more complete understanding of factors that associate with PICU admission (non-ICU hospitalized patients were used as our comparative-control group). PICU admission serves as a valuable proxy for the seriousness of attempt, with only very serious cases of self-harm being admitted. We hypothesized that, since PICUs serve distinct regions, an analysis of the variables extracted from the EMR could assist in developing an understanding of clinical, socioeconomic, geographical [6,7], and temporal (yearly, seasonal, time of day, -of incident) [8], demographics for our region. The results presented herein are intended for PICU staff, pediatricians, social workers, chaplains, suicide researchers, parents, educators, psychologists, those working in adolescent community outreach, and mental health fields.

## 2. Materials and Methods

### 2.1. Site and Population

The study and data collection were conducted at Helen DeVos Children’s Hospital (HDVCH) located in Grand Rapids, MI, after local IRB approval (2018-247-SH/HDVCH). HDVCH PICU is a mixed cardiac surgery and medical intensive care unit with approximately 1600 annual admissions, with over 6000 patient days. Seventeen board certified intensivists provide 24 h coverage for a 24-bed unit, with a maximum capability of 36 critically ill children.

Patients included in our study were between 9 and 18 years of age, admitted to HDVCH between January 2011 to December 2017. Medical records were manually screened retrospectively for possible suicide-related diagnoses and admission keywords (overdose, poisoning, ingestion, intoxication, suicide attempt, or altered mental status). Relevant indications for ICU admission included respiratory failure, significant risk for respiratory failure, depressed mental status, seizures, cardiovascular dysfunction, and risk of arrhythmias. We excluded patients <9 years and >18 years of age, with developmental delays and patients where intent of suicide was difficult to establish.

### 2.2. Variables

Data extracted from HDVCH’s EMR included categorical variables: suicide category (drowning, hanging/strangling, poison, drug overdose, gas, cutting, fall by height), race of patients (African American, Caucasian, Hispanic, other), and type of insurance (unknown, commercial, government), discharge disposition (home, psychiatric/rehab, expired, other). Binary variables included sex (dummy variable: male = 0; female = 1), admission through the emergency department (ED) (Yes = 1/No = 0), hospital death (Yes = 1/No = 0), death (Yes = 1/No = 0); numerical variables included basal metabolic index (BMI), age of the patient (years), and length of stay (LOS), median income (Supplemental Table S1). Severity of illness scores included pediatric risk of mortality III (PRISM III) score [9], and Pediatric Index of Mortality (PIM2) [10], both calculated during the first hours of ICU admission.

Temporal variables were computed based on a patient’s hospital admission date and time. Seasonality was qualified as school session: *either school year or summer vacation*. The months June, July, and August were assumed as general summer vacation months for the study population (School Session = *Summer Vacation*), while the remaining months were considered as school year months (School Session = *School Year*). Furthermore, the day of the week of the hospital admission was used to determine whether the admission occurred on a weekend or a weekday; time of admission was defined as office hours (7:30 a.m.–5:30 p.m.) versus night hours (5:30 p.m.–7:30 a.m.) [11,12].

The patient-level data were supplemented with zip code-aggregated data from the U.S. Census Bureau’s 2017 5-year American Community Survey estimates, and included median household income, various demographic and socioeconomic variables [13].

### 2.3. Statistical Analyses

Quantitative data are expressed as median ± interquartile range. Qualitative data are expressed as frequency (percent). Group comparisons between hospitalized PICU and non-PICU admitted cases for numeric data were implemented using Mann–Whitney U-Tests; qualitative comparisons were made using Fisher’s Exact Tests. A *p*-value of < 0.05 was considered statistically significant.

Modeling the odds of PICU admission was performed using a binomial logistic regression model [14]. With the large skew between PICU and non-PICU admissions, a multivariable model was selected prioritizing model parsimony to prevent over-fitting and complete model separation.

Zip code tabulation areas (ZCTAs) shapefiles were obtained using the tigris package offered in R statistical software [15]. These shapefiles are pulled from the U.S. Census Bureau’s TIGER database. Frequencies for PICU and non-PICU suicide-related admissions were then calculated for each zip code and standardized based on the U.S. Census Bureau’s 2017 ACS estimates of middle school and high school enrollment to calculate the frequency per 1000 students [12]. Additional zip-code level information (rural population, percent of family homes, and housing unit estimates) were obtained through the Integrated Public Use Microdata Series (IPUMS) National Historical Geographic Information System [16]. This information was then used to create bivariate choropleth maps in R [17,18], using median values to classify intensity. Relative PICU admissions were calculated for all ZCTAs as the count of standardized PICU admissions divided by the total combined count of standardized PICU and non-PICU admissions and given as a percent (Supplemental Figures S2–S4). This allowed for an exploratory approach to examine spatial relationships between the observed zip code suicide-related admissions and aggregate-level demographic information. All analyses were performed in R version 3.6.0 [14].

Given the temporal component of the data (suicide-related admissions over time), basic seasonal decomposition was performed using an equidistant moving average and average values at time points to explore seasonality.

## 3. Results

### 3.1. Demographics

**Figure 1:**
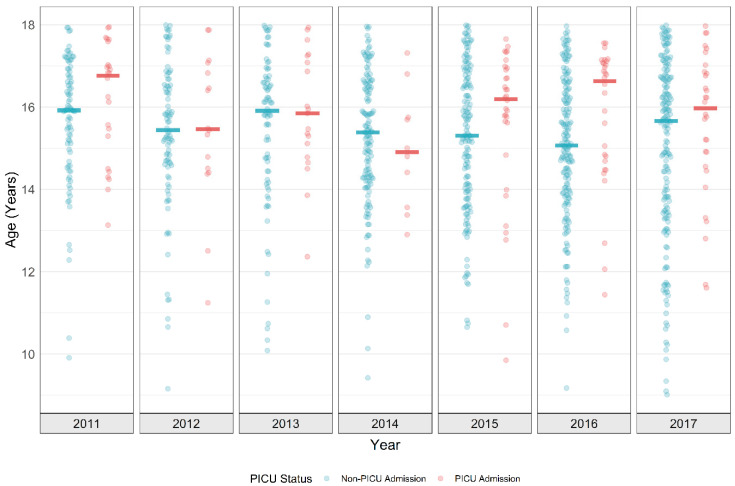
Observed Age Distribution of PICU (pediatric intensive care unit) and Non-PICU Patients (2011–2017).

Patients included in the analysis from 1036 were hospitalized (*n* = 875) and those with a PICU admission (*n* = 161). Initial findings from the demographic analysis revealed statistically significant differences between the patients who were hospitalized versus those admitted to the PICU (Table 1), including age, race, BMI, discharge disposition, LOS, mortality, school session, and time of day of incident. Age was strongly related to PICU admission; median age of PICU-admitted patients being 16 years and non-PICU-admitted patients having a median age of 15.5 years (*p* = 0.001) (Figure 1). While the median age difference is statistically significant, the clinical impact may be viewed as dubious. Figure 1 details the full age distribution for each study-year by admission status. Patients admitted to the PICU have a consistently higher median age, with a more skewed distribution leading to higher densities at older ages. Although not statistically significant, females consistently appeared more frequently in both PICU and non-PICU admissions (64% and 68.6%, respectively). Race/ethnicity yielded significant associations with PICU admissions (*p* = 0.002), as evident in the predominant Caucasian PICU admissions (83%). Patients admitted to the PICU had lower median BMIs (22.0 vs. 23.0, *p* = 0.006). The suicide category did not strongly associate with PICU admissions (*p* = 0.745). A larger number of non-PICU patients were discharged home after their hospitalization (*p ≤* 0.001), spent less time in the hospital (1.6 vs. 1.9; *p ≤* 0.001), and were more likely to survive their hospitalization (*p* = 0.038). In terms of temporality, non-PICU admissions were higher during the school year (*p* = 0.045) and night hours (*p* = 0.044). While many of these covariates were found to be statistically significant, a delineation between statistical significance and clinical significance should be considered when evaluating these findings.

### 3.2. Logistic Regression

A binomial logistic regression was run to predict PICU admission while controlling for other covariates. All relevant covariates were assessed in individual univariate models, and a stepwise selection method was used to create a multivariable model while prioritizing model parsimony by minimizing Akaike Information Criterion (AIC) (Table 2).

The final logit model consisted of race, age, BMI, ED admission, school session, and time of day as covariates predicting PICU admission.

After controlling for all other variables in the model, Black and Hispanic patients were found to have considerably lower odds of a PICU admission when compared to White/Caucasian patients (OR: 0.49 and 0.17, respectively). However, given that much of the local population is identified as White/Caucasian, and this skew is reflected in the sample (over 70% of all patients), the low number of minority patients overall should be placed into context. Moreover, this result also contrasts with other findings [19].

Older patients are significantly more likely to be admitted to the PICU (OR: 1.21, *p* = 0.005), as are patients with smaller body mass indices (OR: 0.95, *p* = 0.0005). Interestingly, with all other multivariable covariates being held constant, initial admission into ED significantly increases the odds of PICU admission (OR: 1.48, *p* = 0.039). This relationship is only marginally significant in the univariate analysis. The time of year relative to school session is also significant, with patient encounters occurring during summer vacation (June–August) having significantly higher odds of being admitted into the PICU relative to patient encounters during the school year (OR: 1.51, *p* = 0.044).

### 3.3. Temporal Changes and Time-Series Decomposition

We were interested in determining the incidence of suicide over the years, over the course of seasons, and broken-down into an even lower denominator of time of day. Additive seasonal decomposition was used to assess the trend of relative PICU admission over time (Supplemental Figure S1). Simple decomposition was implemented by using the moving average to isolate the trend of relative PICU admissions, and a seasonal component was calculated by computing an average value of relative PICU admissions for each month across the entire span of the data (2011–2017). The error of the decomposition was then computed by removing the trend and seasonal components from the original time series data.

The trend for the relative PICU admissions fluctuates, with peaks in 2013 followed by a stark drop-off leading into 2014, and then a rise again in mid-2014. The seasonal components show that relative PICU admissions spike in June and August, and then again in December and January (Supplemental Table S2). These increases seem to correspond to periods in which patients are recently released from, or about to resume school after a holiday or summer vacation.

### 3.4. Spatial Relationships and Patterns

**Figure 2:**
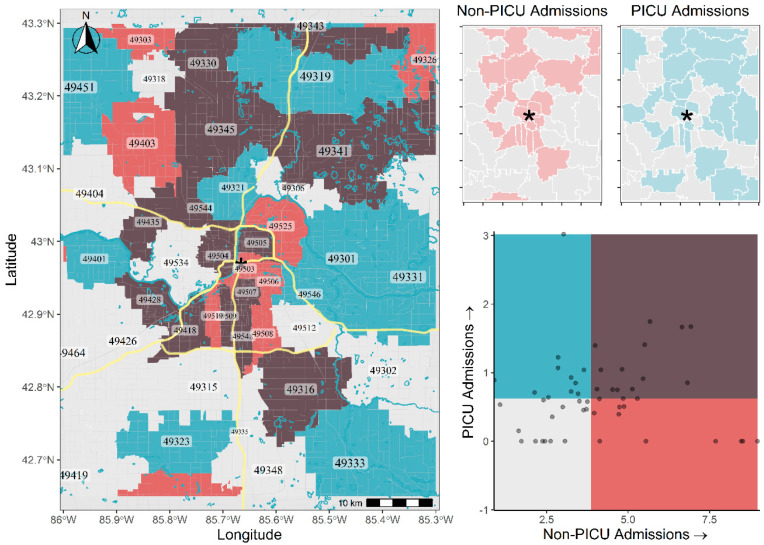
Bivariate Choropleth Map of PICU and Non-PICU Admissions by 5-Digit Zip Code Standardized per 1000 Students.

Spatial relationships were explored by creating bivariate choropleth maps of zip code geographies. Standardizing for a pediatric population size of each zip code tabulation area, a moderately strong correlation in the spatial intensity of PICU admissions and non-PICU admissions was found (Figure 2). The relative PICU admissions also correlate well with aggregate demographic and economic data from the U.S. Census Bureau. Supplemental Figure S2 illustrates higher relative PICU admissions correlating with increasing rural population size (Supplemental Figure S2), with a moderately strong positive correlation present. The percentage increase in housing units from 2011–2017 (an indicator of community change and repurposing) also yields a positive correlation with relative PICU admissions. A similar trend is observed when comparing relative PICU admission to the percentage of family households (Supplemental Figure S4).

To better understand the dynamics of race in our population, Supplemental Figure S5 compares the percentage of patients identifying as White from our sample for each 5-digit zip code and contracts with Census estimate for the percentage of the total population identifying as white. The figure illustrates a general positive correlation between to two measurements, indicating that zip codes with higher white/Caucasian populations produced higher proportions of white/Caucasian patients in our sample. Spatially this trend continues into less-dense population areas.

## 4. Discussion

Suicidal behavior is determined by many factors, none of which are the immediate focus of medical management when the patient is admitted to the PICU. The priorities are more a determination of risk and providing aggressive measures needed to preserve life and minimize morbidity. Once the patient is stabilized in the PICU setting, patients are transferred to the in-patient floor and returned (directly or after a prolonged hospital stay) to a long-term care facility, or to the previous environment (and set of circumstances) that led to the initial behavior. Studies now show that factors such as school systems, socioeconomic and parental unemployment rates are important in determining suicide ideation and attempted and completed suicide in children and adolescents [20,21,22]. In this study, we used the patients in the PICU as the high-risk group and compared them with non-ICU in-patients to extract both clinical and social variables available in the EMR to develop an understanding of SSA that includes community-based risk factors. Furthermore, regardless of whether a suicide attempt results in an ICU admission, the evaluation of risk factors is important and acknowledged by authors.

Caucasian patients had higher admission rates in our PICU (83.2%) compared to other race/ethnic groups. The authors suspect that this is a reflection of racial demographics in Grand Rapids, MI and the Midwest in general, where the Caucasian population is over 82%. Therefore, we question as to whether the number of minority patients in our sample is sufficient as to accurately represent its distribution. Further analysis did show that racial demographics in the sample did positively correlate with the demographics of the zip-code from which the patient address was located, indicating that the sample was at least somewhat representative of the overall population in this perspective (Supplemental Figure S5). Though Caucasians have historically had a higher rate of suicide, there is recent data supporting rising rates of suicide in Black children aged 5–14 [23]. Studies show that Black adolescents have experienced an increase in rates of suicide and lasting injury caused by attempts [5].

In addition, we found BMI to be of significance. Patients in the PICU had significantly lower BMIs than the other patients that were hospitalized for observation. Studies have shown that a higher BMI, though associated with depression, is protective when it comes to SSA [24].

Proxy data in the EMR allowed us to examine the communities of our patients, such as zip codes and payer data. This data, when paired with other databases, such as the U.S. Census Bureau, gives us an understanding of potential social drivers for self-directed violence in our patients.

There are 248 cities in the US with a population of 100–300,000 people making up an eighth of the entire population and this list includes some of the fastest growing cities [25]. Grand Rapids, with a population of 201,000, is one of these cities. We examined the areas around the Grand Rapids and found that there were hotspots that could clearly be identified based on PICU suicide-related admissions. The hotspots were outside the city limits and included regions that are in rapid growth and transition. As the city becomes more urbanized, the regions on the outskirts are subject to unique stressors that need better characterizing. The areas around the Grand Rapids are experiencing some of the greatest growth in population in Michigan. Kent County, which is the County Seat (governmental center) of Grand Rapids, has increased in population at rates of over 9% from 2010 to 2019 [26]. Population increases will change living conditions not only in the cities themselves, but adjoining neighborhoods. Our geospatial examination has shown that suicide increases rapidly in rural and suburban areas compared to urban ones (Supplemental Figure S2). This could simply be a product of a denser pediatric population in urban regions leading to a higher denominator, and thus lower rates, or reflect a scarcity of mental health resources in these areas [3]. Additional steps need to be taken to better understand the geospatial factors around pediatric suicide.

The five-digit zip code tabulation of rural/suburban areas includes large spatial heterogeneity of populations. Future work could entail assessing smaller geographic areas, such as census block-groups to gain a more granular understanding around the immediate environment from which these suicide events emerge. Meyer et al. (2016) used point-pattern modelling with aggregate demographic information obtained from small administrative quarters in Zurich, Switzerland in Europe, to better understand factors associated with psychiatric hospital admissions [27]. This approach could easily be adapted for assessing geographic patterns of suicide-related hospital admissions.

Evidence suggests that schools influence children and adolescent’s health behaviors for self-harm and suicide [28]. However, data exist in the EMR that allow us to examine the influence of the school year on our patients lives. We did this by applying basic time-series decomposition and found that increases in rates of PICU admissions seem to crest when schools are in recess or just about to start. We know that the nature and availability of social ties which are primarily gained from school early on, have high impact on the higher functioning of adolescents. Targeted programs or interventions that originate from schools may alleviate some of the stress these patients feel at transition either from recess or getting back to school. Connecting actual suicide rates in the geographical hotspots to actual school systems is beyond the scope of this paper, as is any commentary on recommended interventions.

Developing an understanding of the communities is important for another reason. The most robust risk factor associated with suicide death is a previous suicide attempt [5]. A suicide attempt is a positive predictor for a completed suicide [29]. Then it becomes important to assess the communities and the support available to this child before returning him or her to that community. There may be a need to review how we think and screen for suicide in terms of age and practice, as it has been found that 45% of suicide patients were seen in a clinical setting in the month prior to their death, versus less than half that number (19%) who were in contact with their mental health providers [30].

If high-risk regions are identified, then active screening by physicians and advanced practitioners in those communities may help alleviate some of the hospital admissions. In a non-ambulatory setting, the Columbia-Suicide Severity Rating Scale (C-SSRS) is the recommended method for suicide screening and risk stratification [31]. These recommendations align with the Joint Commissions National Patient Safety Goal (NPSG.15.01.01) to reduce the risk for suicide [32]. Substantially more people are hospitalized as a result of nonfatal suicidal behavior than are fatally injured, with a substantial number treated in an ambulatory setting [33]. These patients are considered an *at-risk* group but could benefit from being treated as a *high-risk* group to increase the likelihood of receiving early intervention with mental health services [34]. The recommendation for suicide screening practices for patients 10 years of age and older in Western Michigan, according to the Pediatric Behavioral Health Screening Practice Excellence Workgroup of HDVCH, includes (1) in the ambulatory setting, Q9 of the Patient Health Questionnaire-9 (PHQ-9) [35,36]; (2) the Columbia Suicide Severity Rating Scale (C-SSRS) with further assessment and triage stratification—any answer other than “none” is considered a positive screen on Q9 of the PHQ-9.

One limitation of the work is the nature of the experimental design. Future work may include a prospectively collected dataset to avoid any selection bias in the patient population.

## 5. Conclusions

Social determinants of suicide are likely to contribute as much as, if not more than, individual risk factors, but they have been poorly studied to date. This study highlights geospatial and temporal analysis as a means of exploring community risk to develop a better understanding to assist us in providing for the mental health care needs of our communities for future interventions.

## Figures and Tables

**Table 1:** Total Patient Demographics.

Variable	Levels	Missing N	No PICU	PICU	Total	*p*
Total N (%)			875 (84.5)	161 (15.5)	1036	
Age	Median (IQR)	0	15.5 (2.6)	16.0 (2.4)	15.6 (2.5)	0.001
Gender	Female	0	600 (68.6)	103 (64.0)	703 (67.9)	0.271
	Male		275 (31.4)	58 (36.0)	333 (32.1)	
Race	White	0	609 (69.6)	134 (83.2)	743 (71.7)	0.002
	Black		90 (10.3)	10 (6.2)	100 (9.7)	
	Hispanic		86 (9.8)	5 (3.1)	91 (8.8)	
	Other		90 (10.3)	12 (7.5)	102 (9.8)	
BMI	Median (IQR)	116	23.0 (8.0)	22.0 (5.0)	23.0 (7.3)	0.006
ED Admission	Non-ED	0	435 (49.7)	68 (42.2)	503 (48.6)	0.086
	ED		440 (50.3)	93 (57.8)	533 (51.4)	
Discharge Disp.	Home	0	320 (36.6)	31 (19.3)	351 (33.9)	<0.001
	Psychiatric/Rehab		533 (60.9)	121 (75.2)	654 (63.1)	
	Expired		4 (0.5)	3 (1.9)	7 (0.7)	
	Other		18 (2.1)	6 (3.7)	24 (2.3)	
LOS (days)	Median (IQR)	0	1.6 (1.5)	1.9 (1.7)	1.6 (1.6)	<0.001
Insurance Type	Unknown	0	16 (1.8)	1 (0.6)	17 (1.6)	0.681
	Commercial		781 (89.3)	145 (90.1)	926 (89.4)	
	Government		78 (8.9)	15 (9.3)	93 (9.0)	
Suicide Category	Ingestion	0	580 (66.3)	106 (65.8)	686 (66.2)	0.745
	Hanging/Strangulation		7 (0.8)	3 (1.9)	10 (1.0)	
	Suicidal Ideation		95 (10.9)	17 (10.6)	112 (10.8)	
	Other/Self Harm		28 (3.2)	4 (2.5)	32 (3.1)	
	Self-Harm not Apparent		165 (18.9)	31 (19.3)	196 (18.9)	
Hospital Death	Survived	0	871 (99.5)	158 (98.1)	1029 (99.3)	0.080
	Expired		4 (0.5)	3 (1.9)	7 (0.7)	
Death	Survived	0	867 (99.1)	156 (96.9)	1023 (98.7)	0.038
	Expired		8 (0.9)	5 (3.1)	13 (1.3)	
Weekday/Weekend	Weekday	0	662 (75.7)	124 (77.0)	786 (75.9)	0.764
	Weekend		213 (24.3)	37 (23.0)	250 (24.1)	
School Session	School Year	0	674 (77.0)	112 (69.6)	786 (75.9)	0.045
	Summer Vacation		201 (23.0)	49 (30.4)	250 (24.1)	
Time of Day	Night Hours	0	645 (73.7)	106 (65.8)	751 (72.5)	0.044
	Office Hours		230 (26.3)	55 (34.2)	285 (27.5)	
Median Income	Median (IQR)	56 (%)	49,428.0 (18,162.0)	50,232.0 (20,163.2)	49,428.0 (18,160.0)	0.097

Note: Categorical data are expressed as frequency (percent) and analyzed via Fisher’s Exact Test; Numeric data are expressed as median ± interquartile range and analyzed via Mann–Whitney U-Test. *p*-value of < 0.05 was considered statistically significant. BMI: basal metabolic index; Discharge Disp: discharge disposition; ED: emergency department; IQR: inter-quartile range; LOS: length of stay; PICU: pediatric intensive care unit.

**Table 2:** Results of Logistic Regression Analysis for PICU and Non-PICU admissions.

Dependent: PICU Admission		No PICU	PICU	OR (Univariable)	OR (Multivariable)
Age	Mean (SD)	15.2 (1.8)	15.7 (1.7)	1.17 (1.06–1.30, *p* = 0.003)	1.20 (1.08–1.35, *p* = 0.001)
Gender	Female	600 (85.3)	103 (14.7)	-	-
	Male	275 (82.6)	58 (17.4)	1.23 (0.86–1.74, *p* = 0.252)	-
Race	White	609 (82.0)	134 (18.0)	-	-
	Black	90 (90.0)	10 (10.0)	0.50 (0.24–0.95, *p* = 0.049)	0.53 (0.24–1.05, *p* = 0.091)
	Hispanic	86 (94.5)	5 (5.5)	0.26 (0.09–0.60, *p* = 0.005)	0.19 (0.05–0.51, *p* = 0.005)
	Other	90 (88.2)	12 (11.8)	0.61 (0.31–1.10, *p* = 0.120)	0.62 (0.30–1.17, *p* = 0.166)
BMI	Mean (SD)	24.7 (6.3)	23.1 (5.4)	0.95 (0.92–0.98, *p* = 0.004)	0.95 (0.92–0.98, *p* = 0.004)
Insurance Type	Unknown	16 (94.1)	1 (5.9)	-	-
	Commercial	781 (84.3)	145 (15.7)	2.97 (0.60–53.82, *p* = 0.293)	-
	Government	78 (83.9)	15 (16.1)	3.08 (0.56–57.59, *p* = 0.293)	-
Suicide Category	Ingestion	580 (84.5)	106 (15.5)	-	-
	Hanging/Strangulation	7 (70.0)	3 (30.0)	2.35 (0.50–8.58, *p* = 0.222)	-
	Suicidal Ideation	95 (84.8)	17 (15.2)	0.98 (0.55–1.67, *p* = 0.941)	-
	Other/Self Harm	28 (87.5)	4 (12.5)	0.78 (0.23–2.04, *p* = 0.651)	-
	Self-Harm not Apparent	165 (84.2)	31 (15.8)	1.03 (0.66–1.57, *p* = 0.901)	-
Weekday/Weekend	Weekday	662 (84.2)	124 (15.8)	-	-
	Weekend	213 (85.2)	37 (14.8)	0.93 (0.62–1.37, *p* = 0.711)	-
School Session	School Year	674 (85.8)	112 (14.2)	-	-
	Summer Vacation	201 (80.4)	49 (19.6)	1.47 (1.01–2.12, *p* = 0.043)	-
Season	High Season	276 (80.0)	69 (20.0)	-	-
	Low Season	599 (86.7)	92 (13.3)	0.61 (0.44–0.87, *p* = 0.005)	0.62 (0.43–0.91, *p* = 0.013)
Time of Day	Night Hours	645 (85.9)	106 (14.1)	-	-
	Office Hours	230 (80.7)	55 (19.3)	1.46 (1.01–2.08, *p* = 0.040)	1.41 (0.94–2.07, *p* = 0.088)
Median Income	Mean (SD)	51,669.2 (13,855.4)	54,187.8 (16,094.6)	1.00 (1.00–1.00, *p* = 0.044)	-

Note: Covariates in the multivariable model were selected based on a stepwise selection method Number in data frame = 1036, Number in model = 920, Missing = 116, AIC = 767.8, C-statistic = 0.666, Hosmer-Lemeshow = Chi-sq (8) 10.94 (*p* = 0.205). *p*-value of < 0.05 was considered statistically significant. AIC: Akaike Information Criterion; BMI: basal metabolic index; OR: odd ratio; PICU: pediatric intensive care unit; SD: standard deviation.

## Supplementary Materials

The following are available online at https://www.mdpi.com/2227-9067/8/2/59/s1, Figure S1: Time-Series Decomposition of Relative PICU admissions over the course of the Study Period; Figure S2: Higher Relative PICU Admissions Correlating with Increases Rural Population Size; Figure S3: Standardized PICU and non-PICU Admissions Relative to Housing; Figure S4: Standardized PICU and non-PICU Admissions Relative to Family Households. Figure S5: Comparison of Census Population Demographics (Percent White), and observed Race within Study Sample; Table S1: Data Dictionary; Table S2: Median relative frequency of PICU admissions for each month, accompanied by seasonal decomposition.
