# Supplementary material for: Understanding Suicide in Our Community through the Lens of the Pediatric ICU: An Epidemiological Review (2011–2017) of One Midwestern City in the US
Source: Children (Basel). 2021 Jan 20;8(2):59. doi: 10.3390/children8020059 (PMC7909391; doi:10.3390/children8020059)
Supplement: Supplementary file 1 [file children-08-00059-s001.pdf]

children

MDPI

Supplementary Materials:

Figure S1. Time-Series Decomposition of Relative PICU admissions over the course of the Study Period.

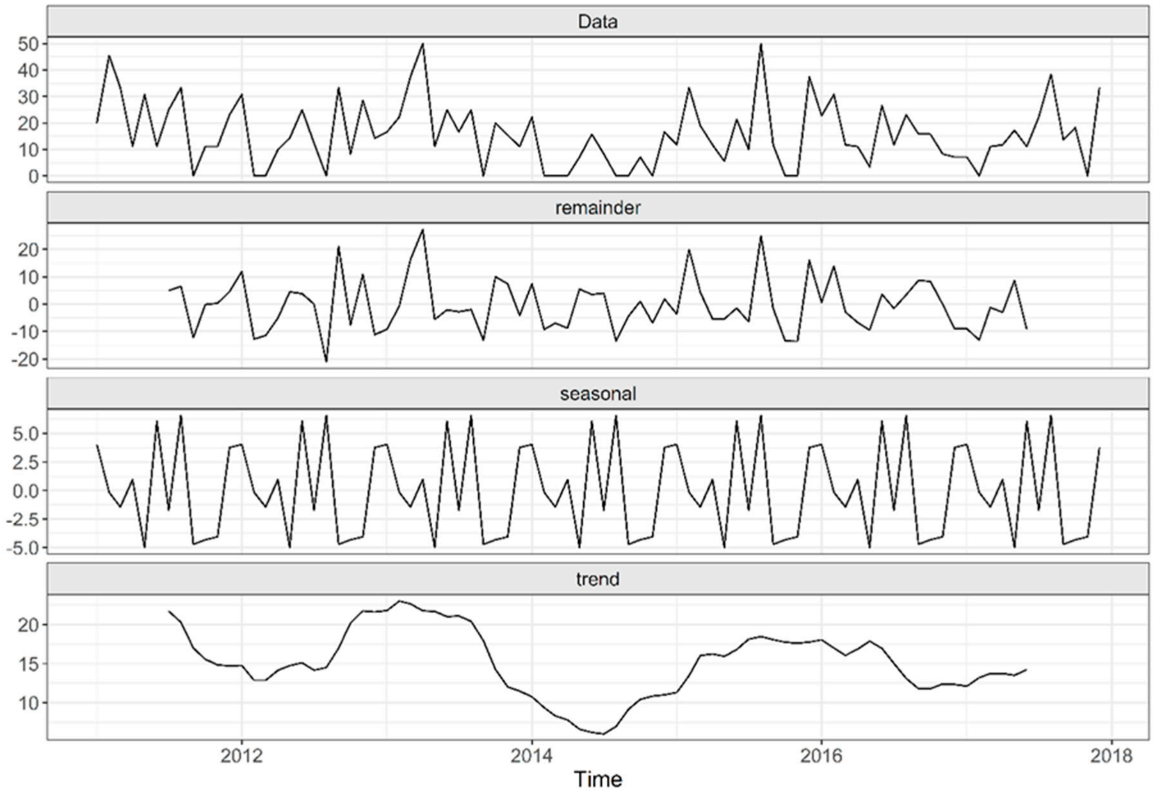

Note: As stated in the Methods section. Time series decomposition consisted of applying a simple seasonal decomposition using an equidistant moving average.

Figure S2: Higher Relative PICU Admissions Correlating with Increases Rural Population Size

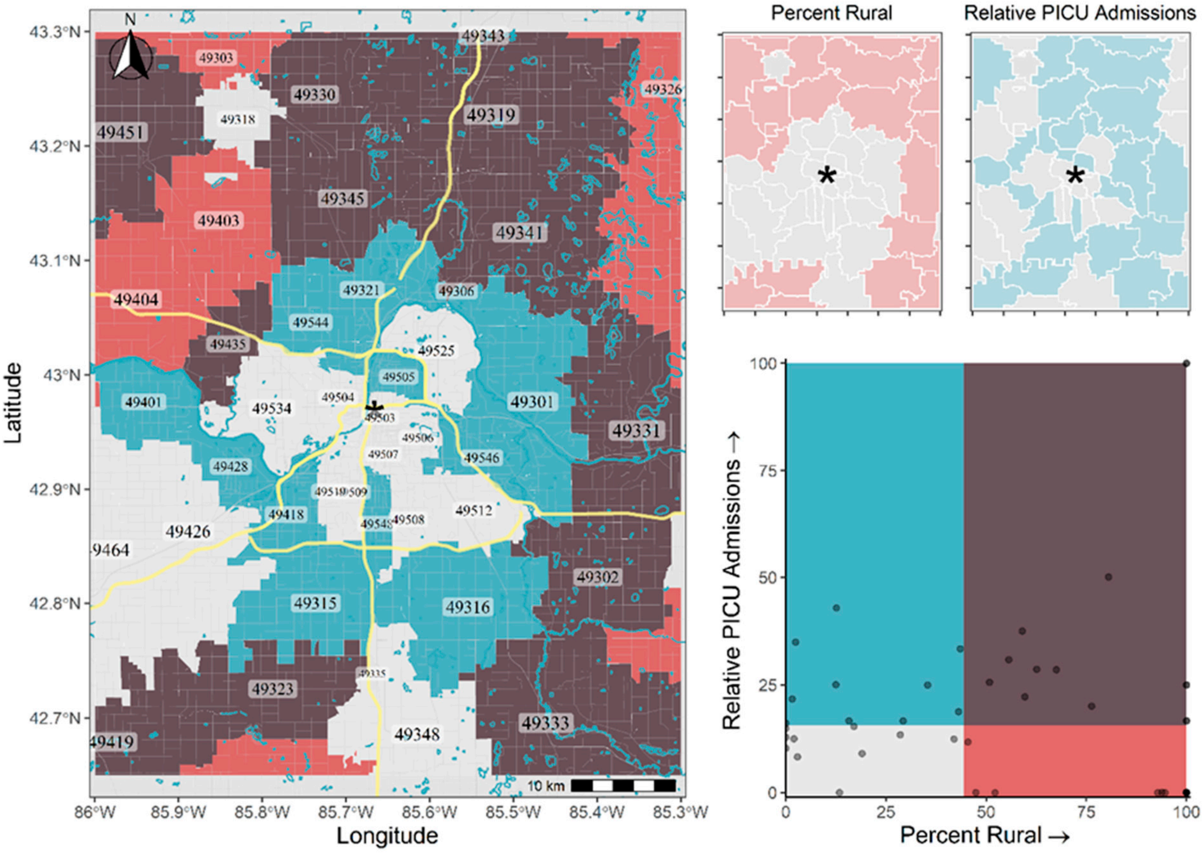

Note: A weak positive correlation between the percent of population defined as rural by the U.S. Census Bureau and the relative number of PICU admissions. Despite being located in an urban center, Helen Devos Children's Hospital has close proximity to a large rural population. Over half of the observed zip codes have a majority rural population. Areas colored in gray or dark red are what we would expect to observe given a perfect positive correlation. Areas in blue have higher relative PICU admissions given their rural population proportion, and areas in red have a lower relative PICU admission considering their rural population proportion.

Figure S3. Standardized PICU and non-PICU Admissions Relative to Housing.

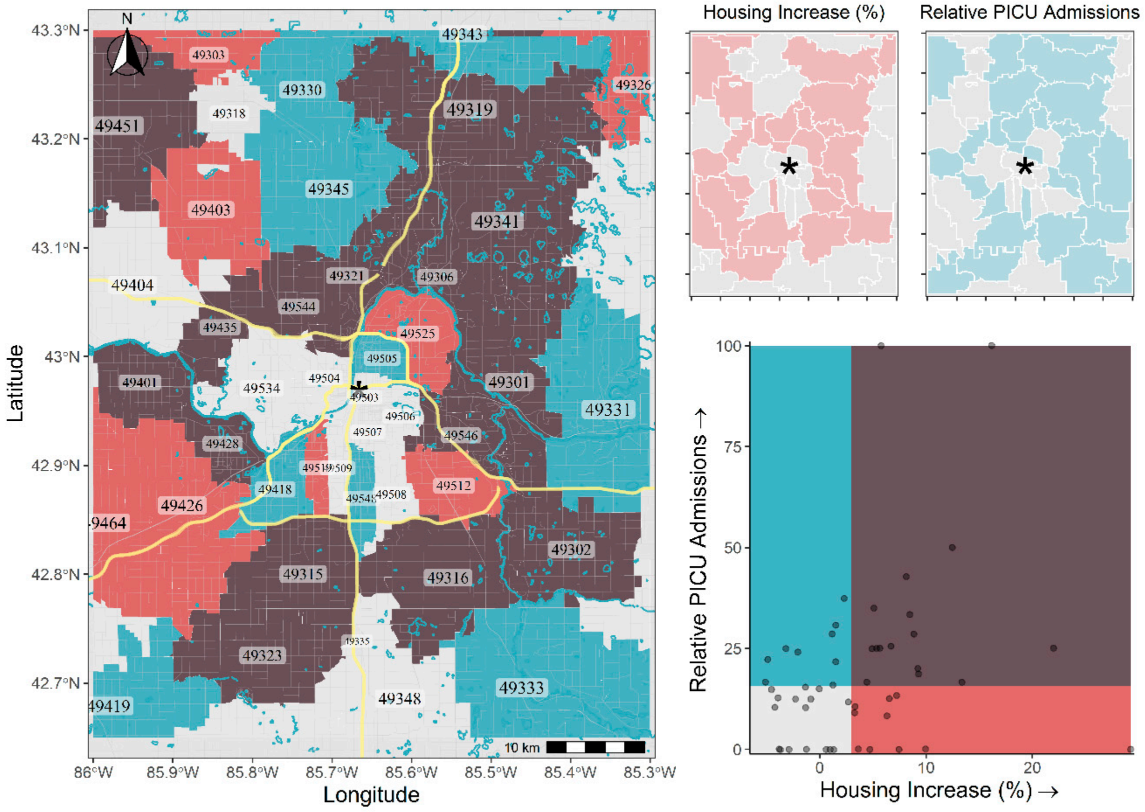

Note: Percent Housing increase from 2011-2017 was used as a proxy variable for community change & development over the study period. A positive correlation is observed when compared against the relative PICU admissions for each zip code, meaning that areas with large increases in housing development also appear to have a higher proportion of suicide-related visits that are serious & life-threatening, resulting in admission to the PICU

Figure S4. Standardized PICU and non-PICU Admissions Relative to Family Households.

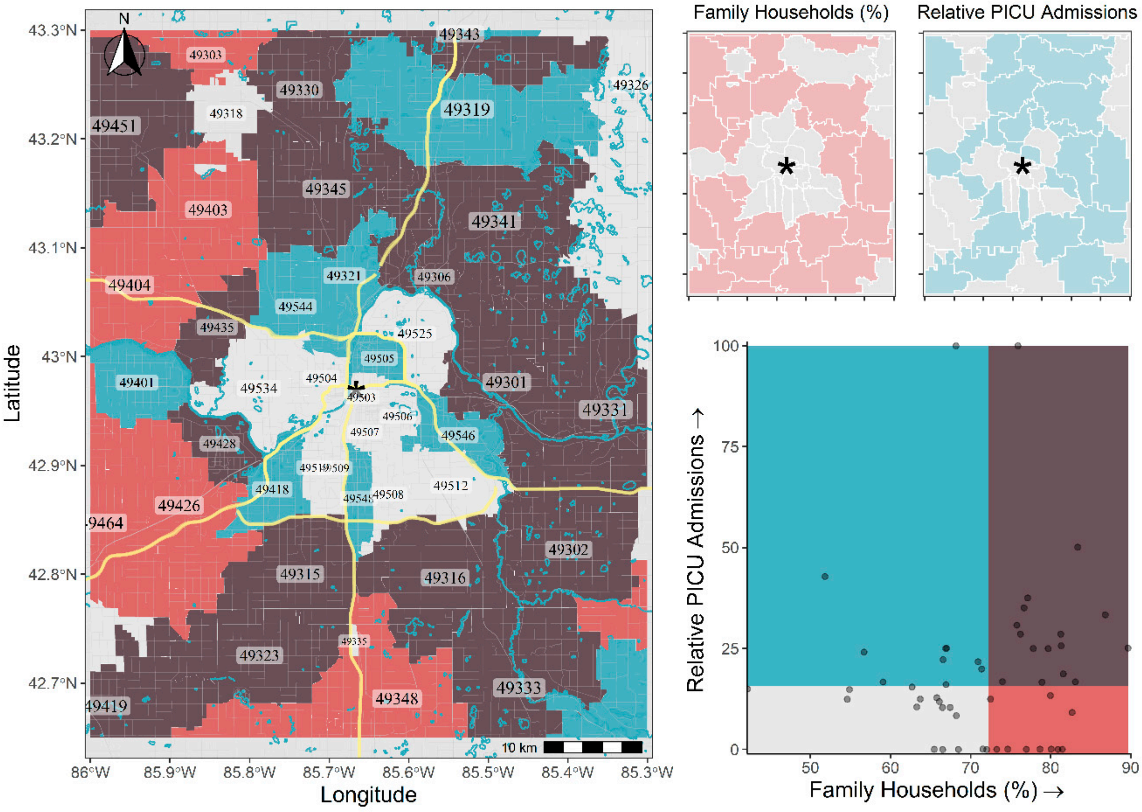

Note: A weak-moderate positive correlation is present comparing the percentage of family households and the relative PICU admissions in a given zip code; zip codes with large percentages of family homes tended to have a higher proportion of suicide-related visits result in PICU admission

Figure S5. Comparison of Census Population Demographics (Percent White) and observed Race within Study Sample.

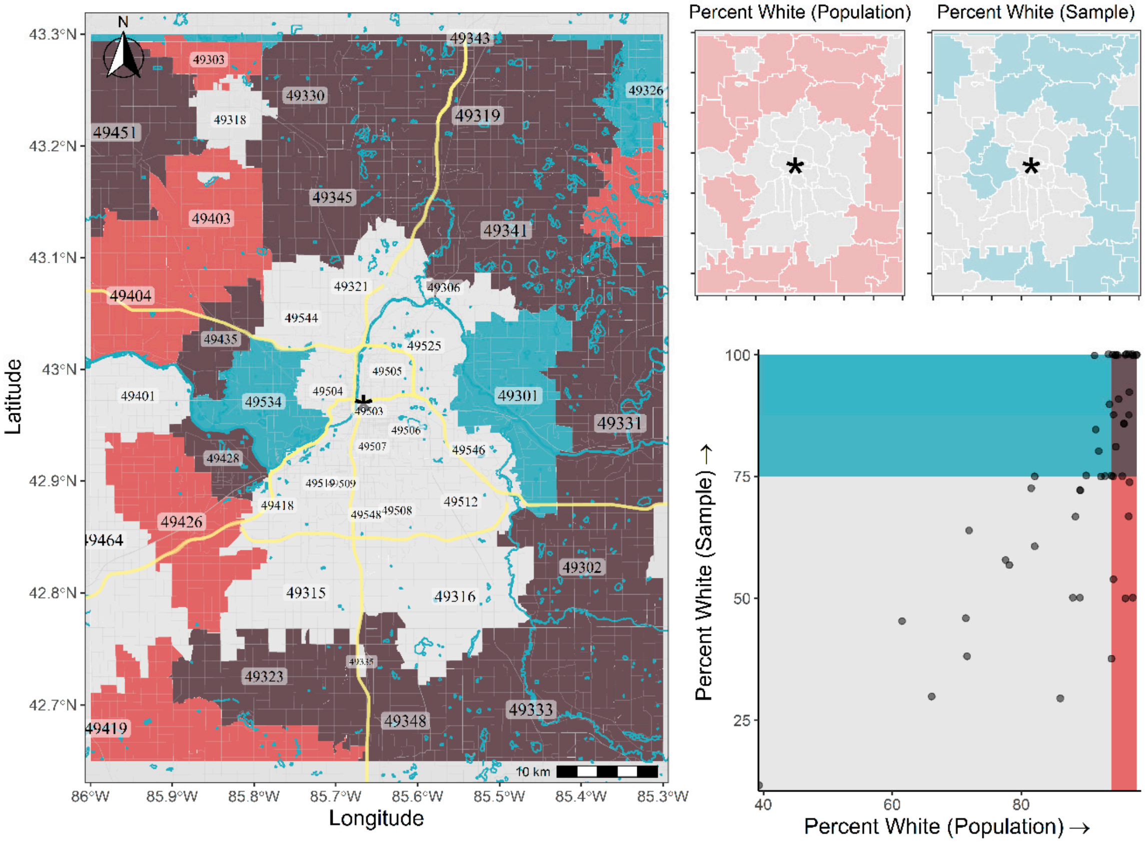

Note: Results show a moderately string positive relationship between the proportion of white/Caucasian individuals in the population (as indicated from the U.S. Census) and the proportion of white/Caucasian patients present in the study sample. As is common, the population becomes increasingly white/Caucasian as distance from the city center (center of map) increases, as does the observed study sample.

Table S1. Data Dictionary.

Variable	Description	Formats
study_id	Unique study id to link to correlation tool	
age_yrs_	Patient age in years at admission	Numeric
gender	Gender of Patient	0=Female; 1=Male
race	Race/Ethnicity of Patient	1=White; 2=Black;3=Hispanic; 4=American Indian/Alaska Native; 5=Asian; 6=Other; 7=Unknown
ins_type	Insurance Type for the Patient	0=Unknown; 1=Commercial; 2=Government
bmi	Body Mass Index of the Patient at Admission	Numeric
med_inc	Median Income within Patient Zip Code	Numeric
avg_inc	Average Income within Patient Zip Code	Numeric
admit_ed	Admitted via ED vs. Non-ED	0=Non-ED; 1=ED
picu	PICU Admission	0=No; 1=Yes
disch_disp	Discharge Disposition	1=Home; 2=Physchiatric/Rehab; 3=Expired; 4=Other
los_days	Length of Stay in Days	Numeric
hosp_death	Hospital Death	0=No; 1=Yes
ip_cnt	Total IP counts after the first encounter for suicide	
ed_cnt	Total ED counts after the first encounter for suicide	
diag1-diag7	Suicide Diagnoses related to Visit	
visit_reas	Reason for Visit	

Table S2. Median relative frequency of PICU admissions for each month, accompanied by seasonal decomposition.

Month	Median Relative Frequency	Seasonal Adjustment
January	20.00	4.03
February	22.22	-0.15
March	11.76	-1.43
April	11.11	0.96
May	11.11	-4.98
June	21.43	6.06
July	12.50	-1.70
August	25.00	6.56
September	11.76	-4.72
October	11.11	-4.32
November	8.33	-4.06
December	16.67	3.76
